# Localized Versus Diffuse Corneal Invasion in Fungal Keratitis: Histological Insights from *Candida albicans* and *Fusarium falciforme*

**DOI:** 10.3390/jof11090688

**Published:** 2025-09-22

**Authors:** Johanna Theuersbacher, Lukas Haug, Alexander Maximilian Aldejohann, Grit Walther, Oliver Kurzai, Daniel Kampik, Jost Hillenkamp

**Affiliations:** 1 Department of Ophthalmology, University Hospital Würzburg, 97080 Würzburg, Germany; 2 Dr. Senckenberg Institute of Pathology, University Hospital Frankfurt, Goethe University Frankfurt, Theodor-Stern-Kai 7, 60590 Frankfurt am Main, Germany; 3 Institute for Hygiene and Microbiology, University of Würzburg, 97080 Würzburg, Germany; 4 National Reference Center for Invasive Fungal Infections, Leibniz Institute for Natural Product Research and Infection Biology-Hans Knoell Institute, 07745 Jena, Germany

**Keywords:** cornea, fungal keratitis, histopathological analysis, keratoplasty, adaptive optics imaging

## Abstract

Fungal keratitis is a severe infection that often requires surgical intervention and is associated with poor outcomes. Penetrating keratoplasty allows for the complete removal of the fungal infiltrate and thus can be a turning point in therapy. The depth of pathogen invasion, which cannot always be reliably assessed by slit lamp examination, can be accurately determined through histological analysis of the corneal trephinate. In this study, we histologically analyzed two corneal trephinates obtained during an emergency keratoplasty performed for uncontrollable mycotic infections. In case 1, caused by *Candida albicans*, the infiltrate remained localized at the site of pathogen entry. In contrast, in case 2, *Fusarium falciforme* demonstrated extensive tissue invasion, spreading destructively throughout the cornea. This invasion pattern suggests that *Fusarium* keratitis is difficult to control due to its aggressive spreading behavior within the tissue. This explains the high rate of penetrating keratoplasty required in such cases.

## 1. Introduction

Fungal keratitis is a serious eye infection that leads to keratoplasty in one third of cases in Germany and even results in enucleation in 10% of cases [1]. The treatment of fungal keratitis cases leaves ophthalmologists with ever-increasing challenges. On the one hand, only a small number of effective medications are available [2,3] and, on the other hand, resistance against the most common agents is frequent [4,5,6]. Conservative treatment is therefore often unsuccessful and penetrating keratoplasty to remove the fungus from the eye is the only option [7,8]. Typically, the affected corneal tissue is excised and replaced with a sterile donor graft.

Data from the German Keratomycosis Registry indicate that *Fusarium* and *Candida species* together account for nearly three quarters of all recorded cases [1]. *Fusarium* spp. are ubiquitous in nature and harbor pathogenic potential well-known for plants and also for humans [5,9]. While *Fusarium* keratitis was formerly common in tropical areas and mostly associated with trauma (inoculation of plant material), the rising use of contact lenses has boosted current infection rates, especially affecting urban areas and moderate climate zones [1,5]. Recent analyses confirm that members of the *Fusarium solani* species complex (FSSC), including *F. falciforme*, predominate among *Fusarium* infections in Germany and that in vitro resistance is typically high [4].

Ecological niches of *Candida* spp. are both environmental and commensal, including human skin and mucosal surfaces [10]. One of the most common risk factors for *Candida* keratitis is a history of prior corneal surgery [11]. *C. albicans* in particular is often associated with human infections ranging from superficial to deep-seated invasive and life-threatening diseases [10]. However, resistance rates are usually low [10].

In this study, we performed histopathologic analysis of two corneal trephinates obtained during penetrating keratoplasty for uncontrollable fungal keratitis. The invasion pattern of [1] *Candida albicans* and [2] *Fusarium falciforme* within the corneal tissue is analyzed and examined for pathogen-specific differences that may have implications for future treatment strategies for fungal keratitis.

## 2. Materials and Methods

### 2.1. Ethical Approval and Patient Consent

Data collection and evaluation for fungal keratitis patients were approved by the Institutional Review Board of the University of Würzburg (no. 62/20_z-sc). Both patients signed informed consent for publication of their clinical histories.

### 2.2. Microbiological Findings

*Candida albicans* was isolated from anterior chamber fluid of patient 1 following incubation in Sabouraud dextrose enrichment broth (SDB). Subcultivation on chromogenic media (CHROMagarTM *Candida*) revealed typical green colonies, which were analyzed by mass spectrometry (MALDI-TOF, VITEK MS, bioMérieux, Craponne, France) leading to final species confirmation.

*Fusarium falciforme* (syn. *Neocosmospora falciformis*) including *F. paranaense* was identified in patient 2 from an eye swab cultured in SBD enrichment medium. After subcultivation a mycelium yielded typical growth formation (felty brown, poorly sporulating) and microscopic examination revealed banana shaped one- to three-celled conidia consistent with *Fusarium* spp. Molecular species identification was achieved by partially sequencing transcription elongation factor 1-alpha (TEF) and RNA Polymerase II second largest subunit (RPB2) loci and led to final species ID. Both sequences are deposited in GenBank under the accession numbers PV605566 (TEF) and PV861767 (rpb2).

Resistance testing was performed by VITEK2 AST, bioMérieux, France, or EUCAST broth microdilution.

### 2.3. Histological Processing

In both cases, the corneal trephinates were bisected immediately after excision. Half of each sample was submitted for pathological evaluation, undergoing routine macroscopic examination, formalin fixation, and paraffin embedding, and hematoxylin–eosin (HE) and periodic acid–Schiff (PAS) stains were performed. *Candida* sections were additionally stained with Grocott’s methenamine silver stain. In both cases, histological analysis revealed a variable inflammatory infiltrate with, in parts, very few identifiable mycotic structures.

## 3. Results

### 3.1. Case Reports

Patient 1 (female, 76 years) underwent elective Descemet membrane endothelial keratoplasty (DMEK) due to Fuchs endothelial dystrophy predominantly in the right eye (day 0) at an external center. She was admitted to the ophthalmology department with a postoperative corneal infiltrate unresponsive to topical antibiotics (day 43). Given the clinical suspicion of fungal keratitis, the anterior chamber was irrigated twice (day 44 and 46) and intracameral amphotericin B was administered (50 µg/mL in 0.1 mL). *Candida albicans* was confirmed by microbiological culture from the first anterior chamber punctate (day 61). Despite systemic therapy with fluconazole (400 mg daily loading dose, 200 mg daily maintenance dose, administered from day 43 to day 110) and hourly amphotericin B (0.5%) eye drops, the infiltrate remained refractory. Emergency full-thickness keratoplasty was performed on day 47, combined with intracameral amphotericin B administration (50 µg/mL in 0.1 mL). On day 51, a vitrectomy with intravitreal amphotericin B (50 µg/mL in 0.1 mL) was performed. For the remaining follow-up period of over 3 years (up to day 1356), findings were stable without further recurrence of *Candida* keratitis.

Patient 2 (male, 62 years) presented one day after returning from Thailand with pain in the right eye (day 0). Due to suspected fungal keratitis, hourly topical voriconazole (1%) was started. Unfortunately, the corneal infiltrates increased in size so that an anterior chamber puncture with injection of intracameral 2% voriconazole was performed on days 37 and 43. Emergency full-thickness keratoplasty with intracameral injection of voriconazole 2% was performed on day 45 due to increasing intraocular inflammation with hypopyon formation. Antifungal therapy was discontinued on day 119, as postoperative findings were stable. There was no evidence of recurrence including the remaining follow-up period until day 190.

### 3.2. Microbiological Susceptibility Testing

For *Candida albicans*, automated resistance testing (VITEK2 AST, bioMérieux, France) measured low minimum inhibitory concentration (MIC) values for common antifungals (micafungin ≤ 0.06 mg/L, voriconazole ≤ 0.12 mg/L, fluconazole ≤ 0.5 mg/L, amphotericin B ≤ 0.5 mg/L), proving an overall benign susceptibility profile.

For *Fusarium falciforme*, susceptibility testing by EUCAST broth microdilution confirmed reduced in vitro activities of most antifungals (MIC values: amphotericin B 2 mg/L, natamycin 8 mg/L, voriconazole > 8 mg/L, posaconazole > 8 mg/L, itraconazole > 8 mg/L, isavuconazole > 8 mg/L, terbinafine > 32 mg/L). These findings are consistent with the known resistance profile of *Fusarium falciforme*. The fungal isolate of *Fusarium falciforme* is available at the Jena Microbial Resource Collection (JMRC) under the strain number NRZ-2024-0725.

### 3.3. Pattern of Pathogen Invasion

The invasion patterns of filamentous fungi and yeasts were fundamentally different in the two corneal trephinates.

**Figure 1:**
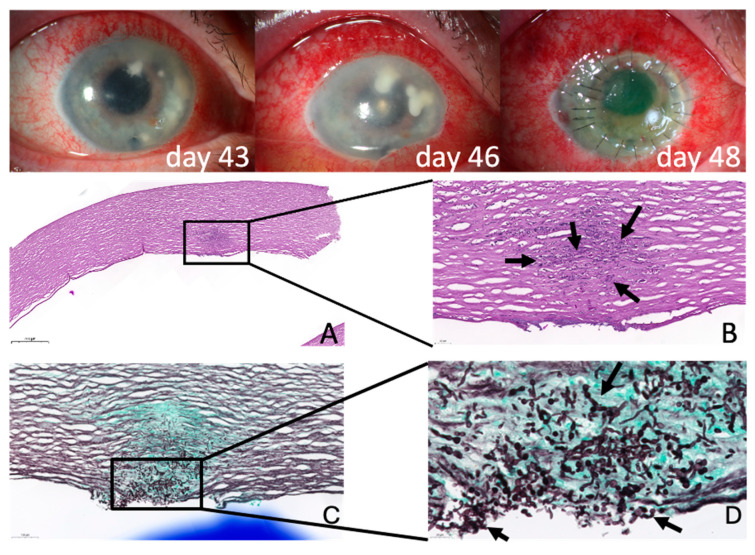
**Late-onset *Candida albicans* keratitis following Descemet membrane endothelial keratoplasty.** Clinical appearance at presentation (day 43 and 46 after initial DMEK surgery) and after emergency full-thickness keratoplasty (day 48). (**A**–**D**) Histological sections of the corneal trephinate stained with (**A**,**B**) Periodic acid–Schiff (PAS) and (**C**,**D**) Grocott’s methenamine silver stain, both demonstrating localized fungal infiltration only around the site of entry marked with black arrows. (**C**,**D**) Fungal structures stained in black. Scale bars in (**A**) 500 µm, (**B**) 50 µm, (**C**) 100 µm, (**D**) 20 µm.

In patient 1 (*Candida albicans* infection), clinical imaging at initial presentation (day 43) and the day before (day 46) and after (day 48) emergency keratoplasty suggested a fungal infiltration deep into the tissue (Figure 1). However, histological analysis of the trephinate revealed a well-circumscribed fungal infiltration localized to the site of entry at the endothelium. The extent of spread was very limited, deeper stromal layers were not affected. The yeast organisms did not penetrate more than one third of the corneal tissue and caused a sparse infiltrate of neutrophil granulocytes with partial cell decay. The stroma was edematous and swollen on the endothelial side, the Descemet membrane was obviously perforated at the site of the infiltrate. The multilayered corneal epithelium and anterior stroma showed no significant abnormalities.

**Figure 2:**
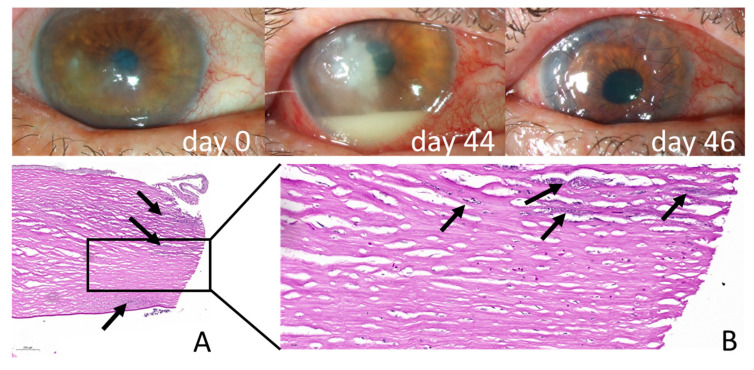
**Late-onset *Fusarium falciforme* keratitis following a stay in Thailand.** Clinical appearance with progressive fungal infiltration and hypopyon formation (days 0 and 44) and after emergency full-thickness keratoplasty (day 46). (**A**,**B**) Histological sections of the corneal trephinate stained with Periodic acid–Schiff (PAS), demonstrating diffuse, full-thickness fungal invasion (black arrows), (**A**) scale bar 200 µm.

In patient 2 (*Fusarium falciforme* infection), slit-lamp microscopy demonstrated an extensive infiltrate into the superficial stroma following epithelial penetration. Histology of the trephinate, however, reveals a trans-corneal invasion by *Fusarium falciforme* with hyphae scattered throughout the whole stroma, which have a particularly high density near the endothelium (Figure 2). This widely spread extent of infiltration indicates diffuse infiltration of the fungus into the complete stromal tissue. 

In both cases, the clinical slit-lamp findings did not reliably reflect the depth of fungal invasion seen histologically.

## 4. Discussion

In this study, two corneal trephinates obtained during emergency full-thickness keratoplasty were histologically analyzed. The two fungal pathogens represent fundamentally different species: *Candida albicans* in patient 1 and *Fusarium falciforme* in patient 2. Although only one specimen was evaluated per case, histological analysis strongly suggests distinct patterns of corneal invasion between yeasts and filamentous fungi. To our knowledge, no systematic histological comparison of the two fungal species in human corneal tissue has yet been published.

Prior reports support the observation that *Candida species* tend to remain localized within the cornea. Wessel et al. [12] described a case of *Candida orthopsilosis* keratitis: the yeast also proliferated very locally in the cornea after DMEK but did not diffusely infiltrate the stroma. We observed the same pattern of tissue invasion in our previously published case of *Candida kefyr (Kluyveromyces marxianus)* following DMEK [7]. Kanavi et al. [13] demonstrated confined posterior stromal localization of *Candida albicans* after deep anterior lamellar keratoplasty (DALK).

This contrasts with the diffuse, full-thickness corneal invasion pattern of *Fusarium falciforme* in patient 2 (Figure 2), highlighting a fundamental difference in tissue behavior between yeasts and filamentous fungi.

This diffuse, aggressive infiltration pattern may also be the reason why *Fusarium* keratitis can hardly be controlled by conservative therapies and often requires surgical interventions, especially keratoplasty [5,14,15]. Although numerous case reports of *Fusarium* keratitis exist [16,17,18,19,20,21], there are hardly any histologic analyses of *Fusarium* keratitis in human corneal trephinates. In two case reports of enucleated eyes with *Fusarium* endophthalmitis, *Fusarium tonkinense* caused a diffuse widespread necrotizing inflammatory reaction in the cornea, anterior chamber angle, and iris [22]. *Fusarium keratoplasticum* led to melting of the corneal tissue to the point of perforation and destroyed the anatomy of the anterior segment, even displacing the lens [23]. Thus, published data further support the notion that *Fusarium species* exhibit more aggressive tissue invasion than *Candida species* in the human cornea.

Based on the slit-lamp findings, the depth of the infiltrate could not be determined with certainty in either case here. In vivo imaging such as confocal microscopy has become increasingly important in recent years and can quickly confirm the diagnosis of a fungal infestation without having to wait for growth in the fungal culture [24,25]. This non-invasive imaging has a high sensitivity and could also be used to assess the depth of the infiltrate. Optical coherence tomography provides cross-sections of the cornea and allows the width of infiltrates and corneal thickness to be measured [26]. It can localize the depth of the infiltrate well and identify endothelial plaques [7,16,27]. Both non-invasive imaging procedures were not used in the two patients but could have provided further information, particularly on the depth of infiltration.

Apart from the different pathogenicity mechanisms of the pathogens, patient-related individual factors could also have influenced tissue invasion. The interval between onset of infection and keratoplasty was comparable in both cases (47 vs. 45 days). However, the mechanism of infection differed fundamentally. *Candida albicans* most likely entered the eye through the surgical procedure, whereas in the case of *Fusarium falciforme* the mechanism cannot be precisely determined. Trauma-related inoculation with plant material during travel to Thailand was suspected, consistent with the known ecology of *Fusarium falciforme* as a plant pathogen, particularly in Southeast Asia [28,29,30]. Thus, the focus of the infection was endothelial in patient 1 and epithelial in patient 2.

Interestingly, one might have assumed that an epithelial-based infection (patient 2) would be more accessible to topical antifungal therapy than an endothelial-based infection (patient 1). However, the clinical outcomes contrasted with this assumption. *Candida albicans* remained localized despite its deeper entry site, whereas *Fusarium falciforme* exhibited widespread stromal invasion despite anterior localization. This observation supports the hypothesis that filamentous fungi possess a greater intrinsic capacity for tissue invasion compared to yeasts. However, only a comparative histological analysis with more samples can conclude in a description of a general biological phenomenon.

## 5. Conclusions

This comparative histological analysis highlights distinct patterns of corneal invasion by *Candida albicans* and *Fusarium falciforme*. While *Candida* infections tended to remain localized, *Fusarium* exhibited aggressive, diffuse stromal infiltration. These findings underscore the importance of early and accurate diagnosis, as well as the potential value of non-invasive imaging techniques to assess the extent of fungal keratitis. Understanding species-specific invasion behavior may help refine therapeutic strategies and improve clinical outcomes in fungal keratitis.

## Data Availability

The original contributions presented in this study are included in the article. Further inquiries can be directed to the corresponding author.

## Abbreviations

The following abbreviations are used in this manuscript:

DALK	Deep anterior lamellar keratoplasty
DMEK	Descemet membrane endothelial keratoplasty
HE	Hematoxylin–eosin
JMRC	Jena Microbial Resource Collection
MIC	Minimum inhibitory concentration
PAS	Periodic acid–Schiff
SDB	Sabouraud dextrose enrichment broth
